# Multi-Grid Reaction-Diffusion Master Equation: Applications to Morphogen Gradient Modelling

**DOI:** 10.1007/s11538-024-01377-y

**Published:** 2024-11-27

**Authors:** Radek Erban, Stefanie Winkelmann

**Affiliations:** 1 Mathematical Institute, University of Oxford, Radcliffe Observatory Quarter, Woodstock Road, Oxford, OX2 6GG UK; 2 Zuse Institute Berlin (ZIB), Takustrasse 7, 14195 Berlin, Germany

**Keywords:** Reaction-diffusion master equation, Multi-grid methods, Morphogen gradient formation, Stochastic simulation algorithms

## Abstract

The multi-grid reaction-diffusion master equation (mgRDME) provides a generalization of stochastic compartment-based reaction-diffusion modelling described by the standard reaction-diffusion master equation (RDME). By enabling different resolutions on lattices for biochemical species with different diffusion constants, the mgRDME approach improves both accuracy and efficiency of compartment-based reaction-diffusion simulations. The mgRDME framework is examined through its application to morphogen gradient formation in stochastic reaction-diffusion scenarios, using both an analytically tractable first-order reaction network and a model with a second-order reaction. The results obtained by the mgRDME modelling are compared with the standard RDME model and with the (more detailed) particle-based Brownian dynamics simulations. The dependence of error and numerical cost on the compartment sizes is defined and investigated through a multi-objective optimization problem.

## Introduction

Compartment-based stochastic reaction-diffusion models have been used for modelling a range of biological processes, including Min-protein oscillations (Fange and Elf 2006) and ribosome biogenesis (Earnest et al. 2015) in *E. coli*, calcium signalling (Denizot et al. 2019; Dobramysl et al. 2016a), gene expression (Winkelmann and Schütte 2016), actin transport in filopodia (Zhuravlev et al. 2010; Erban et al. 2014) and epidemic spreading (Winkelmann et al. 2021). They can be simulated using algorithms for continuous-time discrete-space Markov chains and are mathematically described using the reaction-diffusion master equation (RDME) for the probability mass function (Erban and Chapman 2020; Winkelmann and Schütte 2020). At the analytical level, the RDME builds a bridge between microscopic models for reaction-diffusion processes and macroscopic approximations through partial differential equations (Flegg et al. 2012; Montefusco et al. 2023; Flegg et al. 2015; Kang and Erban 2019).

To formulate a standard compartment-based model, the computational domain is discretized into compartments and diffusion is modelled as a jump process between the compartments (Winkelmann and Schütte 2020). Considering three-dimensional domains, it has been shown that the compartment size cannot be chosen arbitrarily small for systems containing second-order or higher-order reactions, i.e., the error of a compartment-based simulation increases as the compartment size approaches zero (Erban and Chapman 2020). There have been numerous studies (Isaacson 2009; Erban and Chapman 2009; Hellander et al. 2012; Kang et al. 2012; Isaacson 2013; Agbanusi and Isaacson 2014; Hellander and Petzold 2016; Isaacson and Zhang 2018) on the optimal choice of the compartment size and its influence on the approximation quality compared to microscopic modeling approaches given by the Doi model (Doi 1976a, b) or the Smoluchowski model (Andrews et al. 2010; Andrews 2012).

The optimal size of the compartment depends on the diffusion constant (Erban and Chapman 2009; Isaacson 2009; Hellander et al. 2012; Kang et al. 2012; Isaacson 2013; Agbanusi and Isaacson 2014; Hellander and Petzold 2016; Isaacson and Zhang 2018). In particular, if a biological system consists of molecular species with different diffusion constants, the compartment-based model can be naturally generalized to allow for different meshes (compartment sizes) for different chemical species (Cao and Erban 2014; Hellander and Hellander 2020; Li and Cao 2012). In this paper, we analyze such models using a generalization of the RDME which we will call the *multi-grid reaction diffusion master equation* (mgRDME). Numerical simulations of multi-grid reaction-diffusion models allow for high accuracy at reduced numerical cost compared to fully microscopically resolved systems  (Cao and Erban 2014; Chatterjee et al. 2004; Dai et al. 2008). In some computational frameworks (Hellander and Hellander 2020), molecules can transfer from a fine-grained mesh to a coarse-grained mesh whenever appropriate. In the mgRDME that will be analyzed in this work, the grid size remains constant for each species but varies across species.

The mgRDME framework will be analyzed by applying it to biochemical systems of morphogen gradient formation (Saunders and Howard 2009; Kicheva et al. 2007; Bergmann et al. 2007; Gregor et al. 2007; Iyer et al. 2023). Morphogens are signaling molecules whose non-uniform distribution control pattern formation (or morphogenesis) during the development of multicellular organisms. Starting with the pioneering work of Turing (Turing 1952), it has been shown that pattern formation can naturally arise in reaction-diffusion systems through diffusion-driven instability, provided that the system contains at least two chemical species with different diffusion constants (Murray 2002). In particular, stochastic modelling of Turing patterns is a natural application area of the mgRDME framework (Cao and Erban 2014).

In this paper, we will focus on pattern formation (morphogen gradient formation) which results from pre-patterning, i.e., we assume that the studied domain has already been differentiated into two regions and the release of signalling molecules is localized in one of those regions (Rogers and Schier 2011; Shimmi and O’Connor 2003; Shimmi et al. 2005). The formation of morphogen gradients then leads to further pattern formation as the cells recognize and interpret the high or low morphogen concentration (Wolpert et al. 2015). Stochastic models helps us to understand the impact of noise (fluctuations) on the pattern (Teimouri and Kolomeisky 2018; Kolomeisky 2011; Erban and Chapman 2020).

In our model, we distinguish between *signaling molecules* *A*, which are produced locally in a specific spatial region, and *morphogen molecules* *B*, which are produced from *A* through a reaction that can occur anywhere in space. Both species move in space via diffusion. The locally restricted production of *A* (pre-patterning) leads to creation of a morphogen gradient which is then interpreted by cells to produce further patterning (Erban and Chapman 2020, Section 6.8). Since the signaling molecules *A* are assumed to diffuse at a significantly higher rate compared to *B*, our reaction-diffusion systems are well suited to be analyzed via the mgRDME. Moreover, the primary focus lies on the spatial arrangement of the morphogen *B*, represented by its gradient, while the specific spatial arrangement of signaling molecules *A* is of less importance. This prioritization is reflected in the use of distinct compartment sizes for the two species, with a finer resolution dedicated to the morphogen *B*.

We study two reaction networks. In Sect. 2, we consider a reaction-diffusion system with the two chemical species *A* (signal) and *B* (morphogen) being subject to the following three first-order chemical reactions

1 $$\begin{aligned} \emptyset \longrightarrow A \quad \text{(locally } \text{ restricted) } \qquad \text{ and } \qquad A \longrightarrow B \longrightarrow \emptyset \,, \end{aligned}$$

where the empty set $\emptyset$ is interpreted as sources and sinks of molecules (Erban and Chapman 2020), i.e., molecules of signal *A* are continuously produced and converted into molecules of morphogen *B*, which are degraded over time. In Sect. 3, we replace the first-order conversion reaction $A\rightarrow B$ by the second-order *dimerization* reaction $A+A \rightarrow B$, i.e., the chemical reactions (1) are changed to

2 $$\begin{aligned} \emptyset \longrightarrow A \quad \text{(locally } \text{ restricted) } \qquad \text{ and } \qquad A+A \longrightarrow B \longrightarrow \emptyset , \end{aligned}$$

while the diffusion part of the reaction-diffusion model remains the same in both Sects. 2 and 3. Since morphogen gradient systems are effectively one-dimensional (with one ‘important’ direction), we study both systems in a one-dimensional domain. For reaction-diffusion systems with first-order chemical kinetics as in (1), the one-dimensional results are directly applicable in higher dimensions. However, since our second system (2) includes the second-order reaction (dimerization), the diffusion-limited results will depend on the dimension of the physical space (Kang and Redner 1984; Montroll 1969; Ben-Avraham and Redner 1986).

For each of the two reaction networks (1) and (2), we formulate the standard RDME, the mgRDME, as well as a ‘ground truth’ model given by particle-based Brownian dynamics. The compartment-based modeling approaches are compared to the Brownian dynamics results based on the differences in their steady-state distributions. Specifically, we define an error function in terms of the distance between the distribution of morphogen molecules *B* at steady state, and a cost function as a measure of the numerical complexity when simulating the corresponding stochastic processes. Minimizing error and cost over possible choices of compartment sizes for *A* and *B* gives rise to a multi-objective optimization problem, which will be studied for both reaction networks. Our results are further summarized in the discussion Sect. 4. The time-dependent problems are discussed in Appendices 5.1 and 5.4.

## First-Order Fast-Slow Morphogen Gradient Model

In this section, we consider a reaction-diffusion system with the first-order chemical reactions (1). We derive analytic expressions for the long-term spatial distribution of particles, examining the standard RDME, the mgRDME and the Brownian dynamics models in Sects. 2.1, 2.2 and 2.3, respectively. Finally, we compare the models in Sect. 2.4 by means of a multi-objective optimization problem.

**Fig. 1:**
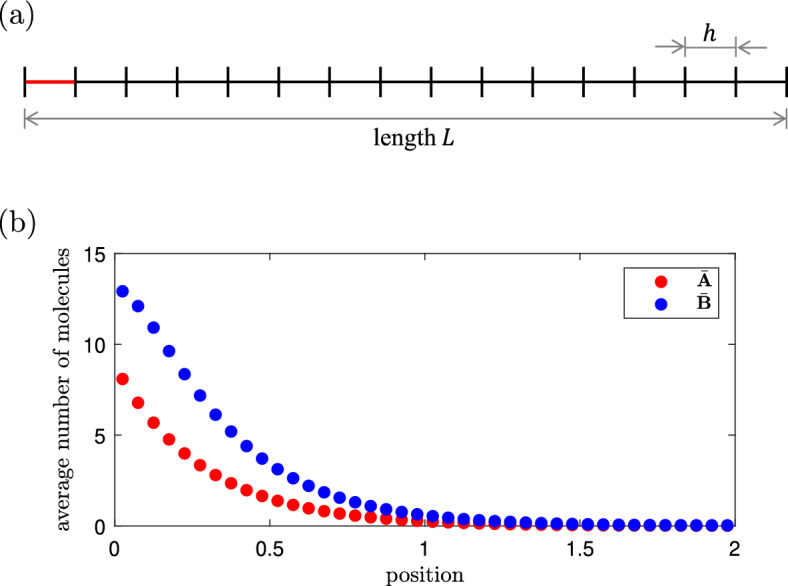
Standard compartment-based model. **a** Schematic of the computational domain [0, *L*] divided into *K* compartments of size *h*. In the first compartment on the left, highlighted in red, molecules of type *A* are produced at rate $$k_1>0$$. **b** The average number of molecules of *A* (red) and *B* (blue) at steady state obtained by solving Eq. (16) for parameters given in (18) and for $$L=2$$ and $$K=40$$ (i.e., we have $$h=L/K=0.05$$, $$D_A/h^2 = 64$$ and $$D_B/h^2 = 4)$$ (Color figure online)

### Standard Compartment-Based Model

We consider molecules of chemical species *A* and *B* that diffuse in the domain [0, *L*], where *L* is the domain length. The diffusion coefficients are denoted $D_A$ and $D_B$, respectively. They have physical units of $\hbox {[length]}^2$/[time]. As for the standard RDME discretization, we divide the domain [0, *L*] into $K = L/h$ compartments of equal length $h>0$. This domain is schematically shown in Fig. 1a. Denoting the chemical species *A* (resp. *B*) in the *i*-th compartment $[(i-1)h,ih)$ by $A_i$ (resp. $B_i$), where $i=1,2,\dots ,K$, then diffusion corresponds to two chains of "chemical reactions" (Cao and Erban 2014; Erban and Chapman 2020):

3 $$\begin{aligned} &  A_1 \; \mathop {{\mathop {\longleftarrow }\limits ^{\displaystyle \longrightarrow }}}^{d_A}_{d_A} \; A_2 \; \mathop {{\mathop {\longleftarrow }\limits ^{\displaystyle \longrightarrow }}}^{d_A}_{d_A} \; A_3 \; \mathop {{\mathop {\longleftarrow }\limits ^{\displaystyle \longrightarrow }}}^{d_A}_{d_A} \; \dots \; \mathop {{\mathop {\longleftarrow }\limits ^{\displaystyle \longrightarrow }}}^{d_A}_{d_A} \; A_K, \end{aligned}$$

4 $$\begin{aligned} &  B_1 \; \mathop {{\mathop {\longleftarrow }\limits ^{\displaystyle \longrightarrow }}}^{d_B}_{d_B} \; B_2 \; \mathop {{\mathop {\longleftarrow }\limits ^{\displaystyle \longrightarrow }}}^{d_B}_{d_B} \; B_3 \; \mathop {{\mathop {\longleftarrow }\limits ^{\displaystyle \longrightarrow }}}^{d_B}_{d_B} \; \dots \; \mathop {{\mathop {\longleftarrow }\limits ^{\displaystyle \longrightarrow }}}^{d_B}_{d_B} \; B_K, \end{aligned}$$

where

5 $$\begin{aligned} d_A = \frac{D_A}{h^2} \qquad \text{ and } \qquad d_B = \frac{D_B}{h^2} \end{aligned}$$

are the jump rates. Since *h* has the physical unit of [length], the jump rates have units $\hbox {[time]}^{-1}$. We assume that molecules of species *A* are produced in the first compartment on the left, highlighted in red in Fig. 1a. This corresponds to the reaction

6 $$\begin{aligned} \emptyset \; \mathop {\longrightarrow }^{k_1/h} \; A_1\,, \end{aligned}$$

where the rate constant $k_1>0$ has units $\hbox {[time]}^{-1}$. Scaling with *h* in reaction (6) is necessary to ensure that after multiplying by the compartment size *h* – as is usual for zero-order reactions (Erban and Chapman 2020) – we get a production rate independent of the compartment size. This gives the same production rate in the first compartment for any value of *h*, and is consistent with our ‘ground truth’ Brownian dynamics model corresponding to partial differential equations (PDEs) (27)–(28) in Sect. 2.3. Since (1) assumes that *A* is converted to *B*, and *B* is degraded in the whole domain, we have the reactions

7 $$\begin{aligned} A_i \; \mathop {\longrightarrow }^{k_2} \; B_i \; \mathop {\longrightarrow }^{k_3 }\;\, \emptyset , \qquad \text{ for } \quad i = 1, 2, \dots , K, \end{aligned}$$

with rate constants $k_2$ and $k_3$ again having physical units of $\hbox {[time]}^{-1}$. The (random) number of particles in the *i*-th compartment at time $t\ge 0$ is denoted by $A_i(t)$ and $B_i(t)$, respectively. Let $p({\textbf{n}},{\textbf{m}},t)$ be the joint probability that $A_i(t)=n_i$ and $B_i(t)=m_i$ for $i=1, 2, \dots , K$, where we use the notation ${\textbf{n}}=[n_1,n_2, \dots , n_K] \in \mathbb {N}^K$ and ${\textbf{m}} = [m_1,m_2,\dots ,m_K] \in \mathbb {N}^K$ to define the system state. To formulate the RDME corresponding to the chemical reaction system (3)–(7), we define operators $\mathcal {O}^+_i, \mathcal {O}^-_i: {\mathbb N}^K \rightarrow {\mathbb N}^K$ by

8 $$\begin{aligned} \mathcal {O}^+_i [n_1, \dots , n_{i-1}, n_i, n_{i+1}, \dots , n_K]:= &  [n_1, \dots , n_{i-1}, n_i + 1, n_{i+1}, \dots ,n_K], \qquad \end{aligned}$$

9 $$\begin{aligned} \mathcal {O}^-_i [n_1, \dots , n_{i-1}, n_i, n_{i+1}, \dots , n_K]:= &  [n_1, \dots , n_{i-1}, n_i - 1, n_{i+1}, \dots ,n_K], \end{aligned}$$

for $i=1,2,\dots ,K$. By means of these operators, we define the diffusion operator $\mathcal {D}:L^1\!\left( \mathbb {N}^K\!\!\times \!\mathbb {N}^K\right) \rightarrow L^1\!\left( \mathbb {N}^K\!\!\times \!\mathbb {N}^K\right)$, where

10 $$\begin{aligned} L^1\!\left( \mathbb {N}^K\!\!\times \!\mathbb {N}^K\right) :=\bigg \{f:\mathbb {N}^K\!\!\times \!\mathbb {N}^K \rightarrow \mathbb {R} \,\bigg |\, \sum _{{\textbf{n}},{\textbf{m}}} f({\textbf{n}},{\textbf{m}}) <\infty \bigg \} \end{aligned}$$

by

11 $$\begin{aligned} \mathcal {D} f({\textbf{n}},{\textbf{m}}):= &  \frac{D_A}{h^2} \sum _{i=1}^{K-1} \Big \{ (n_i+1) \, f (\mathcal {O}^+_i \mathcal {O}^-_{i+1}{\textbf{n}},{\textbf{m}}) - n_i \, f ({\textbf{n}},{\textbf{m}}) \Big \} \nonumber \\ &  + \frac{D_A}{h^2} \sum _{i=2}^{K} \Big \{ (n_i+1) \, f (\mathcal {O}^+_i \mathcal {O}^-_{i-1} {\textbf{n}},{\textbf{m}}) - n_i \, f ({\textbf{n}},{\textbf{m}}) \Big \} \nonumber \\ &  + \frac{D_B}{h^2} \sum _{i=1}^{K-1} \Big \{ (m_i+1) \, f ({\textbf{n}},\mathcal {O}^+_i \mathcal {O}^-_{i+1}{\textbf{m}}) - m_i \, f ({\textbf{n}},{\textbf{m}}) \Big \} \nonumber \\ &  + \frac{D_B}{h^2} \sum _{i=2}^{K} \Big \{ (m_i+1) \, f ({\textbf{n}}, \mathcal {O}^+_i \mathcal {O}^-_{i-1} {\textbf{m}}) - m_i \, f ({\textbf{n}},{\textbf{m}}) \Big \} \end{aligned}$$

and the reaction operator $\mathcal {R}:L^1\!\left( \mathbb {N}^K\times \mathbb {N}^K\right) \rightarrow L^1\!\left( \mathbb {N}^K\times \mathbb {N}^K\right)$ by

12 $$\begin{aligned} \mathcal {R} f({\textbf{n}},{\textbf{m}}):= &  k_1 \Big \{ f (\mathcal {O}^-_1 {\textbf{n}},{\textbf{m}}) - f ({\textbf{n}},{\textbf{m}}) \Big \}\nonumber \\ &  + k_2 \sum _{i=1}^{K} \Big \{ (n_i+1) \, f (\mathcal {O}^+_i{\textbf{n}},\mathcal {O}^-_i {\textbf{m}}) - n_i \, f ({\textbf{n}},{\textbf{m}}) \Big \} \nonumber \\ &  + k_3 \sum _{i=1}^{K} \Big \{ (m_i+1) \, f ({\textbf{n}},\mathcal {O}^+_i {\textbf{m}}) - m_i \, f ({\textbf{n}},{\textbf{m}}) \Big \}. \end{aligned}$$

Then the reaction-diffusion master equation, which corresponds to the system of reactions  (3–7), can be written as follows

13 $$\begin{aligned} \frac{\partial p}{\partial t} ({\textbf{n}},{\textbf{m}},t) = (\mathcal {D} + \mathcal {R}) \, p({\textbf{n}},{\textbf{m}},t) . \end{aligned}$$

The stationary distribution is defined by

$$\begin{aligned} \phi ({\textbf{n}},{\textbf{m}}) = \lim _{t \rightarrow \infty } p ({\textbf{n}},{\textbf{m}},t) \end{aligned}$$

and satisfies the *stationary reaction-diffusion master equation*

14 $$\begin{aligned} 0 = (\mathcal {D} + \mathcal {R}) \, \phi ({\textbf{n}},{\textbf{m}}) . \end{aligned}$$

Solving (14), we obtain the product of Poisson distributions (Jahnke and Huisinga 2007)

15 $$\begin{aligned} \phi ({\textbf{n}},{\textbf{m}}) = \exp \left[ - \sum _{i=1}^K \big (\overline{A}_i + \overline{B}_i\big ) \right] \prod _{i=1}^K \frac{\overline{A}_i^{n_i} \overline{B}_i^{\,m_i}}{n_i! \, m_i!} \, , \end{aligned}$$

where $\overline{A}_i$ and $\overline{B}_i$, for $i=1,2,\dots ,K$, satisfy the steady-state equations

16 $$\begin{aligned} \left( \frac{D_A}{h^2} \, S - k_2 \, I \right) \overline{\textbf{A}} = - k_1 {\textbf{e}}_1, \qquad \left( \frac{D_B}{h^2} \, S - k_3 \, I \right) \overline{\textbf{B}} = - k_2 \overline{\textbf{A}}. \qquad \end{aligned}$$

Here, $I\in {\mathbb R}^{K \times K}$ is the identity matrix and vectors $\overline{\textbf{A}} \in {\mathbb R}^K$, $\overline{\textbf{B}} \in {\mathbb R}^K$, ${\textbf{e}}_1 \in {\mathbb R}^K$ and matrix $S \in {\mathbb R}^{K \times K}$ are given by

17 $$\begin{aligned} \overline{\textbf{A}} = \left( \!\! \begin{array}{c} \overline{A}_1 \\ \overline{A}_2 \\ \overline{A}_3 \\ \vdots \\ \overline{A}_{K-1} \\ \overline{A}_K \end{array} \!\!\right) \!\!, \;\; \overline{\textbf{B}} = \left( \!\! \begin{array}{c} \overline{B}_1 \\ \overline{B}_2 \\ \overline{B}_3 \\ \vdots \\ \overline{B}_{K-1} \\ \overline{B}_K \end{array} \!\!\right) \!\!, \;\; \textbf{e}_1 = \left( \begin{array}{c} 1 \\ 0 \\ 0 \\ \vdots \\ 0 \\ 0 \end{array} \right) \!, \;\; S = \left( \begin{array}{ccccccc} -1 & 1 & 0 & 0 & \dots & 0 & 0 \\ 1 & -2 & 1 & 0 & \dots & 0 & 0 \\ 0 & 1 & -2 & 1 & \dots & 0 & 0\\ \vdots & \vdots & \vdots & \vdots & \ddots & \vdots & \vdots \\ 0 & 0 & 0 & 0 & \dots & -2 & 1 \\ 0 & 0 & 0 & 0 & \dots & 1 & -1 \end{array} \right) \!\!. \end{aligned}$$

The entries $\overline{A}_i$ and $\overline{B}_i$ are the average numbers of molecules of *A* and *B*, respectively, in the *i*-th compartment at equilibrium. The solution $(\overline{\textbf{A}},\overline{\textbf{B}})$ of Eq. (16) is plotted in Fig. 1b for the following choice of rate parameter values

18 $$\begin{aligned} k_1= 100, \qquad k_2 = 2, \qquad k_3 = 1, \qquad D_A=0.16, \qquad D_B=0.01\,, \end{aligned}$$

showing the morphogen gradient. As the stationary distribution $\phi$ is given by a product of Poisson distributions in (15), the values $\overline{A}_i$ and $\overline{B}_i$ not only determine the long-term averages but also the variances of the particle numbers at steady state. Since the values of $\overline{A}_i$ and $\overline{B}_i$ fully characterize the stationary distribution $\phi$, we will compare the models in terms of $\overline{A}_i$ and $\overline{B}_i$.

### Generalized Compartment-Based Model: mgRDME

Assuming that molecules of species *A* diffuse significantly faster than those of species *B* (as indicated by our parameter values in Eq. (18), where $D_A \gg D_B$), we choose a larger compartment size for species *A* (Cao and Erban 2014), while keeping the compartments for species *B* of the same size as in the standard model in Sect. 2.1. Let $h_A=L/K_A$ for $K_A\in \mathbb {N}$ be the compartment length for species *A*, and $h_B=L/K_B$ for $K_B\in \mathbb {N}$, where $K_B>K_A$, be the compartment length for species *B*. For simplicity, we will assume that the ratio $\gamma :=K_B/K_A=h_A/h_B >1$ is a natural number, i.e. $\gamma \in \mathbb {N}$. However, a generalization is possible, see Remark 1 for an explanation. In the mgRDME formulation (Cao and Erban 2014), reaction (6) is replaced by

19 $$\begin{aligned} \emptyset \; \mathop {\longrightarrow }^{k_1/h_A} \;\, A_1, \end{aligned}$$

which implies again that *A*-particles are produced at rate $k_1$ in the first compartment on the left (of the coarser *A*-discretization), independently of the grid size $h_A$. The reaction $A_i \; \longrightarrow \; B_i$ in (7) is generalized to

20 $$\begin{aligned} A_j \; {\mathop {\longrightarrow }\limits ^{k_2/\gamma }} \; B_i, \quad j=1,2,\dots ,K_A, \quad \text{ for } \;\; i \in \mathcal {I}(j) := \{ (j-1)\gamma +1,\, \dots \,, \,j\gamma \}, \end{aligned}$$

which means that after conversion from *A* to *B* the resulting *B*-molecule is placed uniformly in one of the $\gamma$ (smaller) compartments that overlap with the *j*-th (large) compartment of the reacting *A*-particle. The diffusion operator (11) is generalized to

21 $$\begin{aligned} \tilde{\mathcal {D}}f({\textbf{n}},{\textbf{m}}):= &  \frac{D_A}{h_A^2} \sum _{j=1}^{K_A-1} \Big \{ (n_j+1) \, f (\mathcal {O}^+_j \mathcal {O}^-_{j+1}{\textbf{n}},{\textbf{m}}) - n_j \, f ({\textbf{n}},{\textbf{m}}) \Big \} \nonumber \\ &  + \frac{D_A}{h_A^2} \sum _{j=2}^{K_A} \Big \{ (n_j+1) \, f (\mathcal {O}^+_j \mathcal {O}^-_{j-1} {\textbf{n}},{\textbf{m}}) - n_j \, f ({\textbf{n}},{\textbf{m}}) \Big \} \nonumber \\ &  + \frac{D_B}{h_B^2} \sum _{i=1}^{K_B-1} \Big \{ (m_i+1) \, f ({\textbf{n}},\mathcal {O}^+_i \mathcal {O}^-_{i+1}{\textbf{m}}) - m_i \, f ({\textbf{n}},{\textbf{m}}) \Big \} \nonumber \\ &  + \frac{D_B}{h_B^2} \sum _{i=2}^{K_B} \Big \{ (m_i+1) \, f ({\textbf{n}}, \mathcal {O}^+_i \mathcal {O}^-_{i-1} {\textbf{m}}) - m_i \, f ({\textbf{n}},{\textbf{m}}) \Big \}, \end{aligned}$$

while the reaction operator (12) now reads

22 $$\begin{aligned} \tilde{\mathcal {R}}f({\textbf{n}},{\textbf{m}}):= &  k_1 \Big \{ f (\mathcal {O}^-_1 {\textbf{n}},{\textbf{m}}) - f ({\textbf{n}},{\textbf{m}}) \Big \} \nonumber \\ &  + \frac{k_2}{\gamma } \sum _{j=1}^{K_A}\sum _{i\in \mathcal {I}(j)} \Big \{ (n_j+1) \, f (\mathcal {O}^+_j{\textbf{n}},\mathcal {O}^-_i {\textbf{m}}) - n_j \, f ({\textbf{n}},{\textbf{m}}) \Big \} \qquad \nonumber \\ &  + k_3 \sum _{i=1}^{K_B} \Big \{ (m_i+1) \, f ({\textbf{n}},\mathcal {O}^+_i {\textbf{m}}) - m_i \, f ({\textbf{n}},{\textbf{m}}) \Big \}. \end{aligned}$$

Dividing by $\gamma$ in the second line of (22) goes in line with summing over the set $\mathcal {I}(j)$ defined in (20) which contains $|\mathcal {I}(j)|=\gamma$ elements; this can be interpreted as a sum of $\gamma$ reactions $A_j \rightarrow B_i$ that share the rate $k_2$. In total, we obtain the *multi-grid reaction-diffusion master equation* (mgRDME)

23 $$\begin{aligned} \frac{\partial p}{\partial t}({\textbf{n}},{\textbf{m}},t) \,=\, (\tilde{\mathcal {D}} + \tilde{\mathcal {R}}) \,p({\textbf{n}},{\textbf{m}},t) . \end{aligned}$$

Solving the corresponding stationary mgRDME, given by $0=(\tilde{\mathcal {D}} + \tilde{\mathcal {R}})\,\phi ({\textbf{n}},{\textbf{m}})$ in analogy to (14), we obtain a product of Poisson distributions similar to (15):

$$\begin{aligned} \phi ({\textbf{n}},{\textbf{m}}) = \exp \left[ - \sum _{j=1}^{K_A} \overline{A}_j - \sum _{i=1}^{K_B} \overline{B}_i \right] \, \prod _{j=1}^{K_A} \frac{\overline{A}_j^{n_j}}{n_j!} \, \prod _{i=1}^{K_B}\frac{ \overline{B}_i^{\,m_i}}{m_i!}, \end{aligned}$$

where $\overline{A}_j$ and $\overline{B}_i$, for $j=1,2,\dots ,K_A$, $i=1,2,\dots ,K_B$, satisfy the generalized steady-state equations

24 $$\begin{aligned} \left( \frac{D_A}{h_A^2} \, S_A - k_2 \, I_A \right) \overline{\textbf{A}} = - k_1 {\textbf{e}}_1, \qquad \left( \frac{D_B}{h_B^2} \, S_B - k_3 \, I_B \right) \overline{\textbf{B}} = - \frac{k_2}{\gamma } M\overline{\textbf{A}}. \end{aligned}$$

Here, $I_A\in {\mathbb R}^{K_A \times K_A}$ and $I_B\in {\mathbb R}^{K_B \times K_B}$ are identity matrices and vectors $\overline{\textbf{A}} \in {\mathbb R}^{K_A}$, $\overline{\textbf{B}} \in {\mathbb R}^{K_B}$, ${\textbf{e}}_1 \in {\mathbb R}^{K_A}$ and matrices $S_A \in {\mathbb R}^{K_A \times K_A}$, $S_B \in {\mathbb R}^{K_B \times K_B}$ are defined in an analogous manner as before in (17). In addition, there is the block matrix $M\in \mathbb {R}^{K_B \times K_A}$ given by

$$\begin{aligned} M= \left( \begin{matrix}M_1 \\ \vdots \\ M_{K_A} \end{matrix}\right) ,\quad M_j = \left( \begin{matrix} 0 & \dots & 0 & 1 & 0 & \dots & 0 \\ \vdots & & \vdots & \vdots & \vdots & & \vdots \\ 0 & \dots & 0 & 1 & 0 & \dots & 0 \end{matrix}\right) \in \mathbb {R}^{\gamma \times K_A}, \quad j = 1,\dots ,K_A, \end{aligned}$$

with the non-zero entries (i.e., the ones) in the block $M_j\in \mathbb {R}^{\gamma \times K_A}$ placed in the *j*-th column. As in the standard setting, $\overline{A}_j$ and $\overline{B}_i$ give both the long-term mean and variance of the population sizes in the respective boxes, and they fully characterize the stationary distribution $\phi$.

#### Remark 1

The choice of an integer-valued ratio $\gamma$ simplifies notation in (20) and (22). However, a generalization to an arbitrary ratio $K_B/K_A\notin \mathbb {N}$ is straightforward: Let $C_j^{(A)}:=\big [(j\!-\!1)h_A,jh_A\big )$ and $C_i^{(B)}:=\big [(i\!-\!1)h_B,ih_B\big )$ denote the *j*-th and *i*-th compartment of the discretizations chosen for species *A* and species *B*, respectively. Then, reaction $A_j\rightarrow B_i$, meaning that a particle of species *A* located in $C_j^{(A)}$ is converted into *B* located in $C_i^{(B)}$, occurs at rate

25 $$\begin{aligned} \frac{k_2 \, \big |C_i^{(B)}\cap C_j^{(A)}\big |}{\big |C_j^{(A)}\big |}\,, \end{aligned}$$

where $|\cdot |$ represents to the size of the compartment, specifically the length of the interval in the one-dimensional problems considered here. This means that the conversion rate $k_2$ is shared among the potential target compartments according to their overlap with the source compartment.

***Model comparison: standard*** RDME ***versus*** mgRDME***.***

**Fig. 2:**
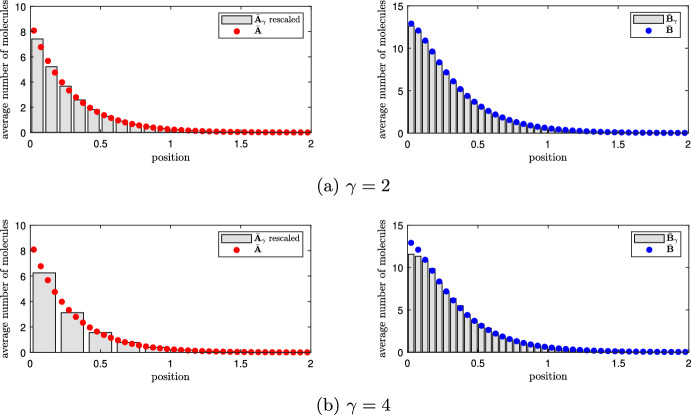
Standard RDME versus mgRDME. Average number of molecules of *A* and *B* at equilibrium obtained by solving the standard stationary RDME (16) (red/blue dots) and by solving the stationary mgRDME (24) (grey bars) for rate constants given in (18) and for $L=2$, $K_B=40$ and **a** $\gamma = 2$, **b** $\gamma = 4$ (Color figure online)

In the following, we denote the solution of the generalized steady-state Eq. (24) by $\bar{\textbf{A}}_\gamma =(\bar{A}^{(\gamma )}_j)_{j=1,2,\dots ,K_A}$, $\bar{\textbf{B}}_\gamma =(\bar{B}^{(\gamma )}_i)_{i=1,2,\dots ,K_B}$ in order to emphasize its dependence on the ratio $\gamma =K_B/K_A$ and to distinguish from the solution $\bar{\textbf{A}}=\bar{\textbf{A}}_1,$ $\bar{\textbf{B}}=\bar{\textbf{B}}_1$ of the standard compartment-based model given by Eq. (16). Figure 2 shows the solution $\bar{\textbf{A}}_\gamma ,\bar{\textbf{B}}_\gamma$ of equation (24) for the same parameter values as in Fig. 1b and for $\gamma =2$ and $\gamma =4$. Instead of $\bar{\textbf{A}}_\gamma$ we plot the rescaled values $\tilde{A}^{(\gamma )}_i:=\bar{A}^{(\gamma )}_j/\gamma$ for $i\in \mathcal {I}(j)$. This is for the purpose of comparability with the solution $\bar{\textbf{A}}$ of the standard steady-state Eq. (16) plotted in Fig. 1b.

**Fig. 3:**
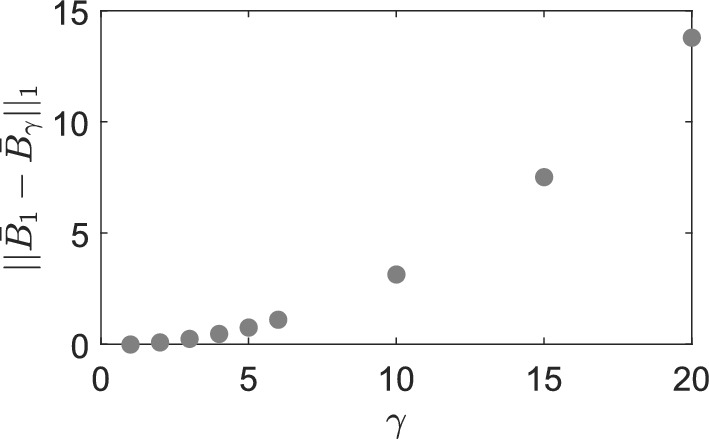
Difference between standard RDME and mgRDME. Difference $\Vert \bar{\textbf{B}}_1-\bar{\textbf{B}}_\gamma \Vert _1$ as a function of the ratio $\gamma =K_B/K_A$ for rate constants given in (18) and for $L=2$, $K_B=60$ and $K_A\in \{60,30,20,15,12,10,6,4,3\}$, where $\bar{\textbf{B}}_1$ the solution of the standard steady-state Eq. (16), and $\bar{\textbf{B}}_\gamma$ the solution of the generalized steady-state Eq. (24) (Color figure online)

We observe a qualitatively good agreement of the generalized solutions with the original one. To see how this agreement depends on the level of coarsening, we plot the difference $\Vert \bar{\textbf{B}}_1-\bar{\textbf{B}}_\gamma \Vert _1$ as a function of $\gamma =K_B/K_A$ in Fig. 3. We choose $\Vert \bar{\textbf{B}}_1-\bar{\textbf{B}}_\gamma \Vert _1$ as the measure of error because the population of species *B* (morphogen) is the output of interest. Note that $\bar{\textbf{B}}_\gamma$ is a vector of fixed size $K_B$ for any $\gamma$, whereas the dimension of $\bar{\textbf{A}}_\gamma$ is $K_A$, which depends on $\gamma$. Consequently, the distance between $\bar{\textbf{A}}_1$ and $\bar{\textbf{A}}_\gamma$ is not directly defined for $\gamma \ne 1$. We see from Fig. 3 that the difference $\Vert \bar{\textbf{B}}_1-\bar{\textbf{B}}_\gamma \Vert _1$ increases monotonically with $\gamma$, which is to be expected since larger values of $\gamma$ represent a higher degree of coarse-graining. However, the increase of the error is nonlinear, and there is an area of values ($\gamma \lessapprox 5$) where the error is sufficiently small to be deemed acceptable. Before we study the approximation quality in more detail, we define in the following section the ‘ground truth’ model given by particle-based Brownian dynamics.

### ‘Ground Truth’ Particle-Based Model: Brownian Dynamics

As our ‘ground truth’ model, we will consider a model with two species *A* and *B* diffusing and reacting in the unbounded domain ${\mathbb R}$ (Erban and Chapman 2020). Molecules of *A* are released at the origin, $x=0,$ with rate $2 k_1$, which has units [$\hbox {sec}^{-1}$]. Due to symmetry, this is equivalent to studying the same model on the half-line $[0,\infty )$ with the rate constant $k_1$ and reflecting boundary conditions at $x=0$ (Erban and Chapman 2007). Molecules of *A* and *B* are diffusing with diffusion constants $D_A$ and $D_B$, respectively. They are subject to the conversion and degradation reactions

26 $$\begin{aligned} A \; \mathop {\longrightarrow }^{k_2} \; B \; \mathop {\longrightarrow }^{k_3 }\;\, \emptyset . \end{aligned}$$

We compare the Brownian dynamics to the simulation on the finite interval [0, *L*], which is discretized into compartments in the RDME and mgRDME frameworks. The effect of the boundary at $x=L$ is discussed in Sect. 5.2 of the Appendix.

Let *a*(*x*, *t*) and *b*(*x*, *t*) be the concentrations of molecules of *A* and *B*, respectively, that is $a(x,t) \, \text{ d }x$ denotes the average number of molecules in the interval $[x,x+\text{ d }x)$ at time *t* in the Brownian dynamics model. Then *a*(*x*, *t*) and *b*(*x*, *t*) satisfy the following PDEs (Kicheva et al. 2007, equation (1))

27 $$\begin{aligned} \frac{\partial a}{\partial t}= &  D_A \frac{\partial ^2 a}{\partial x^2} - k_2 \, a + 2 k_1 \, \delta (x), \end{aligned}$$

28 $$\begin{aligned} \frac{\partial b}{\partial t}= &  D_B \frac{\partial ^2 b}{\partial x^2} + k_2 \, a - k_3 \, b, \end{aligned}$$

where $x \in {\mathbb R}$, and $\delta (x)$ is the Dirac delta function. We consider the boundary conditions

29 $$\begin{aligned} \lim _{x \rightarrow \pm \infty } a(x,t) = \lim _{x \rightarrow \pm \infty } b(x,t) = 0. \end{aligned}$$

Let $\bar{a}(x)$ and $\bar{b}(x)$ be the corresponding stationary distributions, i.e.

$$\begin{aligned} \bar{a}(x) = \lim _{t \rightarrow \infty } a(x,t), \qquad \bar{b}(x) = \lim _{t \rightarrow \infty } b(x,t). \end{aligned}$$

They satisfy the steady-state equations

30 $$\begin{aligned} 0= &  D_A \frac{\text{ d}^2 \bar{a}}{\text{ d } x^2} - k_2 \, \bar{a} + 2 k_1 \, \delta (x) \,, \end{aligned}$$

31 $$\begin{aligned} 0= &  D_B \frac{\text{ d}^2 \bar{b}}{\text{ d } x^2} + k_2 \, \bar{a} - k_3 \, \bar{b} \,, \end{aligned}$$

with boundary conditions

32 $$\begin{aligned} \lim _{x \rightarrow \pm \infty } \bar{a}(x) = \lim _{x \rightarrow \pm \infty } \bar{b}(x) = 0. \end{aligned}$$

We notice that the PDE (27) (resp. the ODE (30)) does not depend on *b*(*x*, *t*) (resp. $\bar{b}(x)$). In what follows, we will therefore first solve the Eq. (27) for the chemical species *A*, and then we will substitute it into the second Eq. (28) for the chemical species *B* to get our ‘ground truth’ solution. In the following, we will do this for the steady-state problem. The time-dependent problem is considered in the Appendix, see Sect. 5.1.

***Steady-state solution***

Since the second derivative of the absolute value function, |*x*|, is equal to $2 \delta (x)$, we can write the solution to Eq. (30) with boundary conditions (32) as

33 $$\begin{aligned} \bar{a}(x) = \frac{k_1}{\sqrt{D_A \, k_2}} \, \exp \left[ -\sqrt{\frac{k_2}{D_A}} \, |x| \right] . \end{aligned}$$

This result is also obtained when taking the limit $t\rightarrow \infty$ of the time-dependent solution *a*(*x*, *t*) as given in Eq. (49) of Appendix 5.1. Substituting into Eq. (31), we get

$$\begin{aligned} D_B \frac{\text{ d}^2 \bar{b}}{\text{ d } x^2} + k_1 \sqrt{\frac{k_2}{D_A}} \, \exp \left[ -\sqrt{\frac{k_2}{D_A}} \, |x| \right] - k_3 \, \bar{b} = 0. \end{aligned}$$

Consequently, using boundary conditions (32), we obtain

34 $$\begin{aligned} \bar{b}(x) &  = \frac{k_1 \sqrt{k_2 \, D_A}}{D_A \, k_3 - D_B \, k_2} \, \exp \left[ - \sqrt{\frac{k_2}{D_A}} \, |x| \right] \nonumber \\ &  \quad \ + \frac{k_1 k_2 \sqrt{D_B}}{(D_B \, k_2 - D_A \, k_3) \sqrt{k_3}} \, \exp \left[ - \sqrt{\frac{k_3}{D_B}} \, |x| \right] \end{aligned}$$

for the concentration $\bar{b}$ of molecules of *B* at equilibrium. Our compartment-based models simulate the problem on the finite interval [0, *L*], with zero-flux boundary condition at $x=L.$ To get the ‘ground truth’ solution (34), we have used the infinite domain, ${\mathbb R}$, with boundary conditions (32). In Sect. 5.2 we show that the effect of non-flux boundary conditions on the ‘ground truth’ solution $\bar{a}$ vanishes for $L\rightarrow \infty$. In particular, the concentration is negligible at $x\ge L$ as long as *L* is large enough, and we can use (33) and (34) for a comparison with the compartment-based models.

### Model Comparison

Since the reaction system under consideration is a first-order reaction network, the standard compartment-based model of Sect. 2.1 converges to the Brownian dynamics solution for $h\rightarrow 0$ (Engblom et al. 2009; Erban and Chapman 2020). Our goal is now to compare Brownian dynamics with the compartment-based model for finite *h*, both for the standard RDME and for the mgRDME. In application, one is mainly interested in the output given by the product *B*, which motivates to define the distance between the models by means of the average number of molecules of *B*.

#### Error Definition

Given the steady-state solution $\bar{\textbf{B}}=(\bar{B}_1,\bar{B_2},\dots ,\bar{B}_{K_B})$ of the mgRDME model with $K_B$ boxes of size $h=L/K_B$ (which agrees with the standard RDME solution when choosing $\gamma =1$), we define the piecewise constant function $\bar{b}_h:[0,L]\rightarrow [0,\infty )$,

35 $$\begin{aligned} \bar{b}_h(x) := \bar{B}_i/h \quad \text{ for } x\in [(i-1)h,ih), \quad i=1,2,\dots ,K_B, \end{aligned}$$

which approximates the steady-state concentration of molecules of *B* as it depends on the location *x*. The $L^1$-distance to the Brownian dynamics solution $\bar{b}$ defines

36 $$\begin{aligned} \text {err}(K_B,\gamma ) := ||\bar{b}-\bar{b}_h ||_{L^1} = \int _0^L |\bar{b}(x)-\bar{b}_h(x)| \, \text{ d }x = \sum _{i=1}^{K_B} \int _{(i-1)h}^{ih} \!\!\!\!|\bar{b}(x)-\bar{B}_i| \, \text{ d }x\,, \qquad \end{aligned}$$

the spatial error in dependence on $K_B$ and on the ratio $\gamma =K_B/K_A$. We note that this error would be positive even if $\bar{b}_h$ was a discretization of $\bar{b}$ (and not the solution of the compartment-based model), simply because of the coarse-graining. We thus expect the error to naturally decrease with decreasing *h*.

**Fig. 4:**
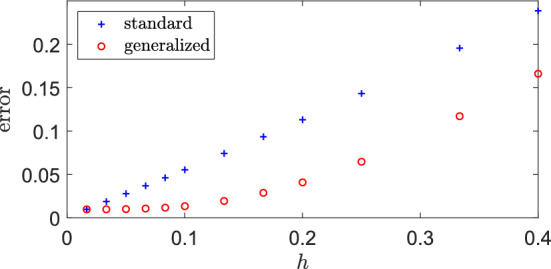
Error in dependence on the compartment size. Error $\text {err}(K_B,\gamma )$, defined in (36), between the steady-state solution $\bar{b}$ of the ‘ground truth’ model given by (34) and its approximation by the standard and the generalized compartment based models with solutions $\bar{b}_h$ defined in (35) in dependence on the compartment size *h*. We have $h_A=h_B=h$ for the standard model, while $h_A=h$ and $h_B=h^*=1/60$ for the multi-grid model. Rate constants given in (18) and $L=2$ (Color figure online)

Figure 4 shows this error in dependence on the grid size *h*, where $h_A=h_B=h$ for the standard RDME, while $h_A=h$ and fixed $h_B=h^*=1/60$ for the generalized mgRDME. For both scenarios, there is a monotone increase of the error with *h*, which is to be expected in a first-order reaction system. The generalized model shows better results as compared to the standard one, which is due to the fixed small grid size $h^*$ for *B*. However, the error $\text {err}(K_B,\gamma )$ seems to be linear in *h* for $\gamma =1$ (standard model, blue crosses), while it is nonlinear in $h=h_A$ for the generalized system and stays close to zero for small $h=h_A$. We can say that for $h=h_A \le 0.1$ ($\gamma \le 6$) the generalized mgRDME model gives a good approximation, while the standard compartment-based model already shows a clear error.

Despite the availability of an analytical solution for the steady-state distribution, there remains a fundamental interest in stochastic simulations of the dynamics, which allow for a more comprehensive exploration of the system’s behavior including complex interactions and rare events. We point out that, in Fig. 4 and for a given *h*, the numerical effort for simulating trajectories is larger for the multi-grid model compared to the standard model due to the retention of the small grid size $h_B=h^*$ for *B*. However, the overall goal of the generalized method is to reduce numerical cost while simultaneously minimizing errors. Therefore, determining an optimal value for $\gamma$ entails multi-objective optimization, a task we will address next.

#### Multi-Objective Optimization

In this section, we study the dependence of the spatial error $\text {err}(K_B,\gamma )$ defined in (36) and of the numerical effort on the grid sizes $h_A$ and $h_B$. We calculate error and cost for different parameter values to show the advantage of the generalized method over the standard method. To define the cost function, we use propensities at steady state because these indicate the runtime of our numerical simulations.

***Propensities at steady state***

The total number of particles at steady state is given by,^1^

37 $$\begin{aligned} \bar{A}_{\text {total}}= \sum _{i=1}^{K_A} \bar{A}_i= \frac{k_1}{k_2}, \qquad \quad \bar{B}_{\text {total}}= \sum _{i=1}^{K_B} \bar{B}_i=\frac{k_2}{k_3}\bar{A}_{\text {total}} = \frac{k_1}{k_3}.\qquad \end{aligned}$$

The overall propensity for a reaction to take place at steady state (no matter which reaction or where in space) is

38 $$\begin{aligned} \bar{r}_{\text {reac}} := k_1 + k_2 \, \bar{A}_{\text {total}} + k_3 \,\bar{B}_{\text {total}} =3\,k_1\,. \end{aligned}$$

Analogously, the overall propensity for a diffusive jump to take place at steady state is

39 $$\begin{aligned} \bar{r}_{\text {diff}}&:= \frac{D_A}{h_A^2}(2 \bar{A}_{\text {total}} - \bar{A}_1-\bar{A}_{K_A}) + \frac{D_B}{h_B^2} (2\bar{B}_{\text {total}} -\bar{B}_1-\bar{B}_{K_B})\nonumber \\&\lessapprox 2\frac{D_A}{h_A^2}\bar{A}_{\text {total}} +2\frac{D_B}{h_B^2} \bar{B}_{\text {total}} \; = \; 2\frac{D_A}{h_A^2}\frac{k_1}{k_2} +2\frac{D_B}{h_B^2} \frac{k_1}{k_3} \end{aligned}$$

with the negative terms in the first line resulting from the fact that diffusive jumps at the outer boundaries of the domain can only go in one direction. The latter expression is a good approximation as long as the population sizes in the outer boxes are not too large (as compared to the total population size), which is the case in the scenarios considered.

**Fig. 5:**
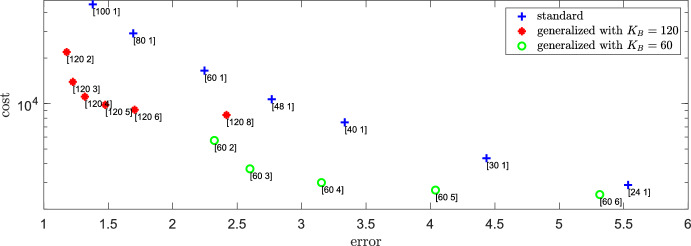
Error and cost depending on the method. Error $\text {err}(K_B,\gamma )$ as defined in (36) and numerical cost $c(K_B,\gamma )$ as defined in (40) for different values of $K_B$ and $\gamma =K_B/K_A$ (given by labels of the form $[K_B\; \gamma ]$). The colored symbols are for orientation: blue pluses for the standard RDME, red stars for the generalized mgRDME with $K_B=120$ and green circles for the generalized mgRDME with $K_B=60$. Parameters are given in (18) and $L=2$ (Color figure online)

The number of iterations steps needed to create a trajectory of the dynamics (starting in steady state, using the Gillespie algorithm (Gillespie 1977, 2007)) scales with $\bar{r}_{\text {reac}}+\bar{r}_{\text {diff}}$. This motivates to define the cost function as a sum of (38) and (39), giving

40 $$\begin{aligned} c(K_B,\gamma ) := 3 \, k_1 + 2 \, \frac{D_A}{h_A^2} \frac{k_1}{k_2} + 2\, \frac{D_B}{h_B^2} \frac{k_1}{k_3}, \end{aligned}$$

where $h_B=L/K_B$ and $h_A=\gamma h_B$. Figure 5 shows the values of error and cost depending on $K_B$ and $\gamma$ (where $\gamma =1$ corresponds to the standard RDME model). We see that the generalized model clearly outperforms the standard model: The Pareto front exclusively comprises points derived from the generalized model. Consequently, the decrease in spatial resolution for species *A* introduced by the generalized model minimally impacts the error in the *B*-solution, relative to the alteration in numerical cost resulting from this coarse-graining. We note the special role of the value $\gamma =4=\sqrt{D_A/D_B}$ which can be considered a good starting point for a practical implementation of the mgRDME as it balances the jump rates of the two species and enhances the model’s efficiency.

##### Remark 2

We note that the scenario would change if we were to define an error metric that incorporates the distance within the *A*-population. This is evident because $||\textbf{A}-\bar{\textbf{A}}(K_B,\gamma )||_1$ rises with $\gamma$, irrespective of $K_B$.

In the examined first-order reaction system, only the production of *A* depends on location, whereas the other reactions occur independently of the molecules’ spatial position. A stronger effect of the grid size on the reaction dynamics is expected for a second-order reaction system, which is studied next.

## Fast-Slow Morphogen Gradient with Dimerization

We consider the fast-slow morphogen gradient system of Sect. 2, replacing the conversion reaction $A\rightarrow B$ by the second-order reaction of *dimerization* $A+A \rightarrow B$, i.e., the chemical reactions (1) are changed to (2), while the diffusion part of the reaction-diffusion model remains consistent with that described in the previous section. Again, we choose the one-dimensional domain [0, *L*]. The associated modeling approaches are introduced in Sect. 3.1 and compared in Sect. 3.2.

### Modeling Approaches for the Dimerization System

Considering the compartment-based models, the diffusion operators are the same as in Sect. 2, i.e., for the standard RDME model it is given by $\mathcal {D}$ as defined in (11), and for the generalized mgRDME model it is given by $\tilde{\mathcal {D}}$ as defined in (21). In contrast, the reaction operators now explicitly depend on the grid size *h* via the second-order reaction $A+A\rightarrow B$. For the standard compartment-based model, given by the RDME $\frac{\partial p}{\partial t}({\textbf{n}},{\textbf{m}},t) = (\mathcal {D} + \mathcal {R}) \, p({\textbf{n}},{\textbf{m}},t)$, it reads

$$\begin{aligned} &  \mathcal {R} f({\textbf{n}},{\textbf{m}}) \,:= \, k_1 \Big \{ f (\mathcal {O}^-_1 {\textbf{n}},{\textbf{m}}) - f ({\textbf{n}},{\textbf{m}}) \Big \}\nonumber \\ &  \qquad + \frac{k_2}{h} \sum _{i=1}^{K} \Big \{ (n_i+2)(n_i+1) \, f (\mathcal {O}^+_i\mathcal {O}^+_i{\textbf{n}},\mathcal {O}^-_i {\textbf{m}}) - \max \{n_i(n_i-1),0\} \, f ({\textbf{n}},{\textbf{m}}) \Big \} \nonumber \\ &  \qquad + k_3 \sum _{i=1}^{K} \Big \{ (m_i+1) \, f ({\textbf{n}},\mathcal {O}^+_i {\textbf{m}}) - m_i \, f ({\textbf{n}},{\textbf{m}}) \Big \} \nonumber \end{aligned}$$

for $h=h_A=h_B$. The rate constant $k_2$ now has physical units [length]/[time], i.e., units of the ‘one-dimensional volume’ divided by time, because $k_2$ is the rate constant of a bimolecular reaction, in contrast to $k_2$ in (12), which has physical units $\hbox {[time]}^{-1}$. Dividing by *h* in the second line stems from the standard scaling of a second-order reaction rate by the volume of the domain in which it takes place, which is given by *h* for the one-dimensional case under consideration. Similarly, for the generalized mgRDME model, we have

$$\begin{aligned}&\tilde{\mathcal {R}}f({{\textbf {n}}},{{\textbf {m}}}) \,:= \, k_1 \Big \{ f (\mathcal {O}^-_1 {{\textbf {n}}},{{\textbf {m}}}) - f ({{\textbf {n}}},{{\textbf {m}}}) \Big \} \nonumber \\ &\;\; + \frac{k_2}{h_A\gamma } \sum _{j=1}^{K_A}\sum _{i\in \mathcal {I}(j)} \Big \{ (n_j+2)(n_j+1) \, f (\mathcal {O}^+_j\mathcal {O}^+_j{{\textbf {n}}},\mathcal {O}^-_i {{\textbf {m}}}) - \max \{n_j(n_j-1),0\} \, f ({{\textbf {n}}},{{\textbf {m}}}) \Big \} \nonumber \\ &\;\; + k_3 \sum _{i=1}^{K_B} \Big \{ (m_i+1) \, f ({{\textbf {n}}},\mathcal {O}^+_i {{\textbf {m}}}) - m_i \, f ({{\textbf {n}}},{{\textbf {m}}}) \Big \} \nonumber . \end{aligned}$$

The factor $1/\gamma$ in the second summand results from splitting the reaction $A+A\rightarrow B$ occurring in the *j*-th compartment into $|\mathcal {I}(j)|=\gamma$ possible ones, depending on the placement of the product *B*:

41 $$\begin{aligned} A_j+A_j \; {\mathop {\longrightarrow }\limits ^{k_2/\gamma }} \; B_i, \quad j=1,2,\dots ,K_A, \quad i \in \mathcal {I}(j) := \{ (j-1)\gamma +1,\, \dots \,,j\gamma \}, \end{aligned}$$

which conforms with the first-order case, see (20) and (22).

***’Ground truth’ particle-based model: Brownian dynamics***

As the ’ground truth’ we choose particle-based dynamics given by the Doi or $\lambda$-$\varrho$ model (Doi 1976a, b; Erban and Chapman 2009; Lipkova et al. 2011). Particles move in space by Brownian motion, and two particles of species *A* undergo dimerization $A+A \rightarrow B$ at rate $\lambda >0$ whenever being within a separation $\varrho >0$, called the reaction radius.

***Relation between rate constants for bimolecular reactions***

Let *h* denote the grid size of the compartment-based model (given by the standard RDME), and let $k_2/h^d$ (where *d* is the dimension of physical space) denote the rate for two molecules of *A* to react when located in the same compartment. The (standard) RDME may be seen as a formal approximation of the Doi model (Isaacson 2008), but it loses second-order reactions as $h\rightarrow 0$  (Isaacson and Peskin 2006; Isaacson 2009; Erban and Chapman 2009). In Isaacson (2013) a *convergent* RDME has been developed, which allows second-order reactions of molecules located in different compartments and thereby does not lose bimolecular reactions as $h\rightarrow 0$. The *standard* RDME may be interpreted as an asymptotic approximation of the *convergent* RDME for $\varrho /h \ll 1$ (Isaacson 2013), where $\varrho$ is the reaction radius of the Doi model. In this case of a comparatively large compartment size, particles can be expected to only react when being located in the same box, and an adequate choice of the binding rate constant $k_2$ is given by $k_2=\lambda |B_{\varrho }|$, where $|B_{\varrho }|$ denotes the volume of the *d*-dimensional sphere of radius $\varrho$. For our one-dimensional domain [0, *L*] (i.e, $d=1$) we thus choose $k_2= 2 \, \varrho \, \lambda$ for the reaction-rate constant of the compartment-based model with sufficiently large compartment size *h*. Note that, on the other hand, *h* has to be small, $h\ll L$, to ensure an appropriate level of spatial resolution (Erban and Chapman 2020).

### Model Comparison

**Fig. 6:**
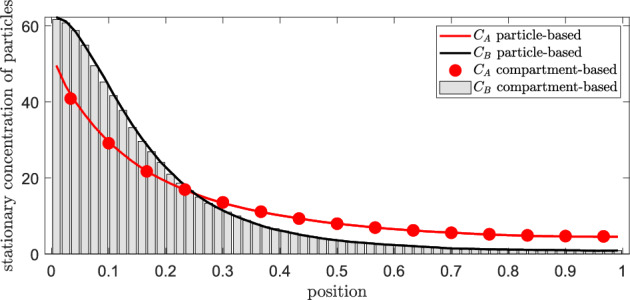
Steady-state solution for the morphogen system with the dimerization reaction. Comparison between Brownian dynamics (solid lines) and generalized mgRDME model (dots/ bars) for dimerization system (2). The parameters are given in (42), together with $L=1$, $K_A=15$ and $K_B=60$ (Color figure online)

In contrast to the first-order reaction-diffusion system of Sect. 2, analytical insights for the dimerization system are relatively limited, so our studies solely rely on computational simulations. Figure 6 shows the steady-state gradients of molecules of *A* and *B*, estimated from long-term stochastic simulations (using the Gillespie stochastic simulation algorithm for the generalized mgRDME and temporal discretization for the Brownian dynamics model). The parameters are chosen as

42 $$\begin{aligned} k_1=50, \;\, \lambda = 5, \;\, \varrho =0.02, \;\, k_2=2 \, \lambda \, \varrho = 0.2, \;\, k_3=2, \;\, D_A=0.16, \;\, D_B=0.01. \; \end{aligned}$$

We observe a close agreement of the generalized compartment-based model with the particle-based dynamics for $K_A=15$ and $K_B=60$. In contrast, for the standard RDME the agreement is worse, as we will see in the next section.

#### Multi-Objective Optimization

**Fig. 7:**
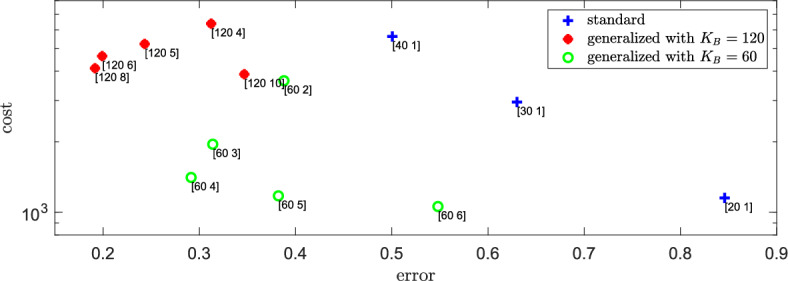
Error and cost for dimerization. Cost $c(K_B,\gamma )$ as defined in (43) and error $\text {err}(K_B,\gamma )$ given by (36) for different values of $K_B$ and $\gamma =K_B/K_A$ (given by labels of the form $[K_B \; \gamma ]\,)$. The parameters are given in (42) and $L=1$. The results are calculated as averages over long-term simulations of length $T=5000$ (Color figure online)

For the spatial error in the distribution of molecules of *B*, we define the function $\text {err}(K_B,\gamma )$ in analogy to (36), that is, we choose the $L^1$-distance between the steady-state solutions of the compartment-based model and of the particle-based model. Let $\bar{A}_{\text {total}}$ and $\bar{B}_{\text {total}}$ be the total number of molecules of *A* and *B*, respectively, of the compartment-based dimerization system at steady state. In contrast to the first-order reaction system of Sect. 2, we lack analytical expression for these quantities and can only estimate them via long-term numerical simulation. The cost function is defined similarly to (40), using the total propensity for diffusion and reaction events at steady state:

43 $$\begin{aligned} c(K_B,\gamma ) := 2\,\frac{D_A}{h_A^2}\bar{A}_{\text {total}} + 2\,\frac{D_B}{h_B^2}\bar{B}_{\text {total}} + k_1 +k_2\,\bar{A}_{\text {total}} (\bar{A}_{\text {total}} -1) + k_3\bar{B}_{\text {total}}\,, \end{aligned}$$

which is quadratic in the total number of molecules of *A* at steady state. Figure 7 illustrates the error and cost values across different combinations of $K_B$ and $\gamma$. We see that the error is *non-monotone* in $\gamma$, which differs from the behavior seen in Fig. 5 for the first-order reaction-diffusion system. It seems that there is an *optimal* $K_A \approx 15 =120/8 =60/4$, where the error in the spatial distribution of molecules of *B* is minimal. Next, we study the relationship between the parameter $K_A$ and the error in the *A*-population.

#### Error Analysis for Species *A*

Given the steady-state solution $\bar{\textbf{A}}=(\bar{A}_1,\bar{A}_2,\dots ,\bar{A}_{K_A})$ of the generalized mgRDME model for the dimerization system, we define

44 $$\begin{aligned} \bar{a}_{h_A}(x) := \bar{A}_i/h_A \quad \text{ for } x\in [(i-1)h_A,ih_A), \quad i=1,2,\dots ,K_A, \end{aligned}$$

as an approximation of the position-dependent steady-state concentration. Let further $\bar{a}(x)$ be the steady-state solution of the particle-based Brownian-dynamics dimerization model (these functions can be approximately determined via numerical simulation). We define the spatial error in *A* in analogy to (36):

45 $$\begin{aligned} \text {err}_A(K_A) := ||\bar{a} -\bar{a}_h||_{L^1} = \int _0^L |\bar{a}(x)-\bar{a}_h(x)| \, \text{ d }x. \end{aligned}$$

Moreover, we consider the difference in the total number of molecules of *A* at steady state:

46 $$\begin{aligned} \text {err}_{\text {total}}(K_A) := \left| \bar{A}_{\text {total}} - \int _0^L \bar{a}(x) \, \text{ d }x\right| . \end{aligned}$$

**Fig. 8:**
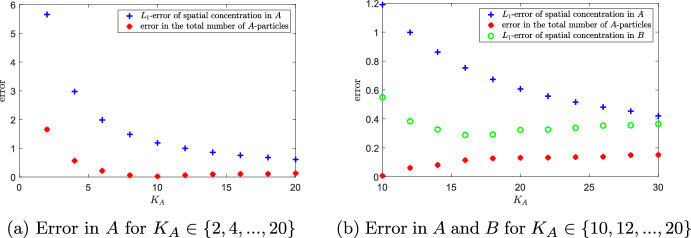
Error in dependence on $K_A$**.** **a** Spatial error $\text {err}_A(K_A)$ given by (45) (blue) and difference $\text { err}_{\text {total}}(K_A)$ in the total number of molecules of *A* defined in (46) (red) for different values of $K_A$. **b** zoom-in, additionally contains the spatial error in *B*, $\text {err}(K_B,\gamma )$ defined in (36), for fixed $K_B=60$ and $\gamma =K_B/K_A$. Parameters given in (42), $L=1$. Long-term simulation of length $T=10^5$ (Color figure online)

As opposed to the first-order system, this quantity can here be non-zero. Figure 8 shows a non-monotone relationship between the number $K_A$ of compartments and the error $\text {err}_{\text {total}}(K_A)$ in the total number of molecules of *A*. The non-monotony is due to a too small grid size $h_A$ for large $K_A$ which contrasts with the condition $\varrho /h \ll 1$ necessary for a good approximation of second-order reactions, see also the paragraph on the relation between the rates on page 17. The spatial error $\text {err}_A(K_A)$ in *A*, however, decreases monotonically with increasing $K_A$ because a higher level of spatial resolution dominates the effect of losing second-order reactions for the parameter values under consideration. The error in *B* (also depicted in Fig. 8b) results from a combination of the two errors in *A* and minimizes for $K_A\approx 16$, which is in consistency with the observations from Fig. 7. The standard compartment-based model would mean to choose $K_A=K_B=60$, inducing a comparatively large error both in the steady-state distribution of molecules of *B* and in the total number of molecules of *A*.

We conclude that the generalized mgRDME model clearly outperforms the standard RDME model for the fast-slow morphogen gradient system with dimerization. For practical implementation, the preliminary choice of $\gamma =\sqrt{D_A/D_B}$ must be carefully adapted to ensure the adequate reproduction of the second-order reaction $A+A \rightarrow B$. Specifically, the compartment size $h_A$ should be chosen based on the reaction radius $\varrho$ and the rate constant $\lambda$ of the particle-based Brownian dynamics model, in conjunction with the diffusion constant $D_A$, but independently of the spatial resolution for *B*. This stands in clear contrast to the first-order chemical reaction system discussed in Sect. 2, where it is more appropriate to adjust the compartment sizes of the two species relative to each other.

##### Remark 3

(Second-order moments for dimerization) Unlike the first-order reaction-diffusion model discussed in Sect. 2, the variance of the particle numbers does here not align with the mean. Consequently, one could also take the variance as a value for comparing the models and defining an error. A numerical comparison of mean and variance for the different model types in application to the dimerization system is given in Appendix 5.3.

## Discussion

We examined the mgRDME as a generalized compartment-based stochastic model for reaction-diffusion kinetics, allowing adapted compartment sizes for different chemical species involved in the system (Cao and Erban 2014). When employed in the context of morphogen gradient formation, the mgRDME is useful because the signaling molecules *A* typically diffuse at a significantly higher rate than the morphogen molecules *B*, and this diffusion rate is a deciding factor for the choice of compartment sizes. In comparison to the ‘ground truth’ particle-based Brownian dynamics simulations, the mgRDME outperforms the standard RDME in terms of accuracy and reduction in numerical cost, both for the first-order reaction network (1) and for the considered dimerization system (2).

Interestingly, however, the structural dependence of error and cost on the compartment sizes is different for the two settings. In Sect. 2, the optimal ratio between grid sizes hinges on the relationship between the diffusion rates. Conversely, in the system involving second-order dimerization in Sect. 3, an optimal grid size for species *A* can be identified (in dependence on its diffusion constant) regardless of the grid size allocated to *B*. In particular, the approximation error may be reduced for the dimerization system by choosing a smaller compartment size for *B* while maintaining the fixed optimal compartment size for *A* undergoing dimerization. This goes in line with the observations for standard RDMEs, where very small grid sizes result in the elimination of the second-order reactions in three-dimensional simulations. The mgRDME circumvents this issue by allowing larger compartment sizes for species involved in second-order reactions. At the same time, the increase in numerical cost caused by the higher spatial resolution is limited, since it is selectively applied solely to the slow species.

Our work shows that the mgRDME is a valuable framework for studying reaction-diffusion systems with multiple scales in diffusion speed of involved molecules. The multi-grid approach enables the mgRDME to more effectively accommodate the inherent conditions of the system under consideration, surpassing the adaptability of the standard RDME. The models with different diffusion constants naturally take into account variations in the sizes of simulated biomolecules, which can range from small calcium ions to relatively large vesicles in neurotransmission dynamics (Ernst et al. 2022, 2023), or from relatively small *G*-actin monomers to larger *F*-actin filaments in simulations of actin dynamics (Zhuravlev et al. 2010; Dobramysl et al. 2016b). Other applications of mgRDME include pattern formation based on Turing instability which requires different diffusion constants (Turing 1952).

In this work, we have solely considered dynamics in one-dimensional domains – a restriction which is well justified in the context of applications to developmental biology, where a modeller is interested in the pattern formation along one body axis, see, for example, dorsal-ventral patterning studies of the Drosophila embryo (Shimmi and O’Connor 2003; Shimmi et al. 2005). However, the ideas behind mgRDME can be extended to higher-dimensional models, where unstructured meshes are used to discretize complex geometries of biological problems to better resolve curved inner and outer boundaries than by Cartesian meshes (Engblom et al. 2009; Isaacson and Zhang 2018). The mgRDME framework can use different unstructured meshes to describe different chemical species. In two-dimensional domains, voxels are typically triangles or squares, while in three-dimensional domains, they are tetrahedra or cubes (Engblom et al. 2009; Earnest et al. 2015). The reaction rates in mgRDME are constructed based on the shared area or volume between voxels from different grids, as described in Remark 1, where we note that voxels may partially overlap, see Eq. (25). The relative size of different meshes (parameterized by $\gamma$ in this paper) can be chosen to optimize the number of spatial diffusive jumps for species with varying diffusion constants. Additionally, different species can be modeled at different resolutions to improve simulation accuracy, with voxel sizes optimized to enhance the description of bimolecular reactions. In applications where high accuracy is only needed in a subset of the simulation domain (Dobramysl et al. 2016a, b), unstructured meshes also offer the advantage to refine the mesh in the corresponding subset of the computational domain. In particular, to model complex biological systems, the mgRDME approach (of using different meshes for different chemical species) can be combined with using different mesh resolutions (for the same chemical species) in different parts of the computational domain (Kang and Erban 2019).

While our analysis has been based on comparison of steady-state morphogen gradients, another way to compare the RDME and mgRDME could be by comparing the first collision and mean reaction times in these models (Li et al. 2018), which can be achieved by analyzing the random walks on lattices (Montroll 1969). Also a comparison of time-dependent solutions (in contrast to steady-state distributions investigated here) is another interesting topic for future research on mgRDME models of morphogen gradients (Saunders and Howard 2009). Some approaches to compare the dynamics prior to steady state are discussed in Appendix 5.4.

## Figures and Tables

**Fig. 9:**
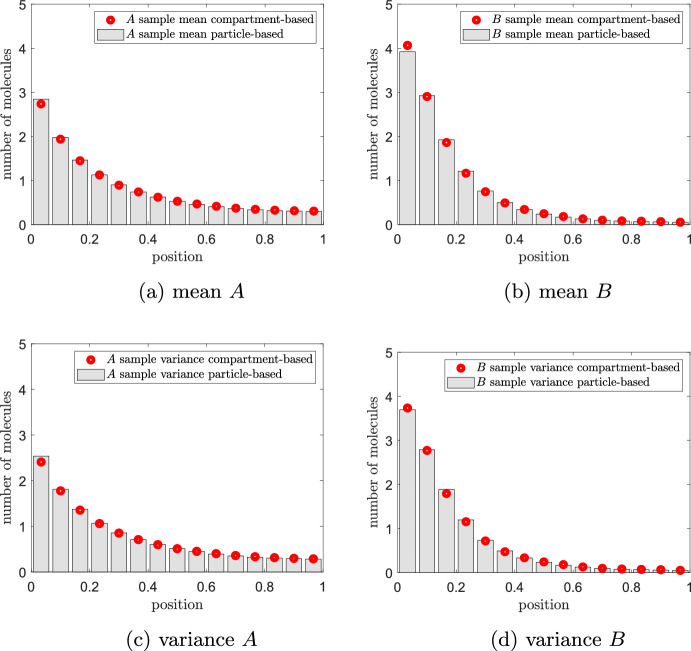
Comparison of mean and variance for dimerization. Mean/variance of the number of molecules of **a**/**c** species *A* and **b**/**d** species *B* at steady state estimated by long-term simulation $$(T=2000)$$ of the compartment-based model (red circles) and of the particle-based Brownian dynamics model (grey bars). Rate constants given in (42), $$L=1$$, $$K_A=K_B=15$$ $$(\gamma =1$$, $$h=1/15)$$ (Color figure online)

**Fig. 10:**
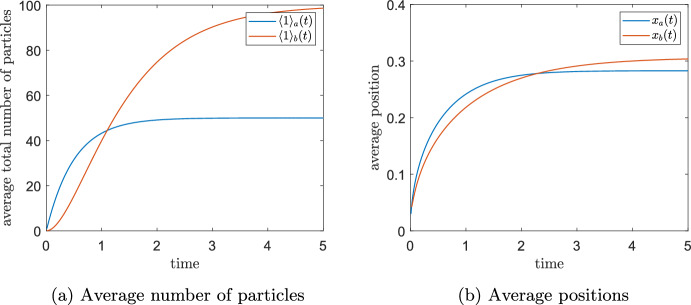
‘Ground truth’ dynamics prior to steady state. **a** Average total number of particles $\langle 1 \rangle _a(t)$ and $\langle 1 \rangle _b(t)$ given by (59) and (61), respectively. **b** Average positions $x_a(t)$ and $x_b(t)$ depending on time given by (60) and (62). Rate constants given in (18) (Color figure online)

**Fig. 11:**
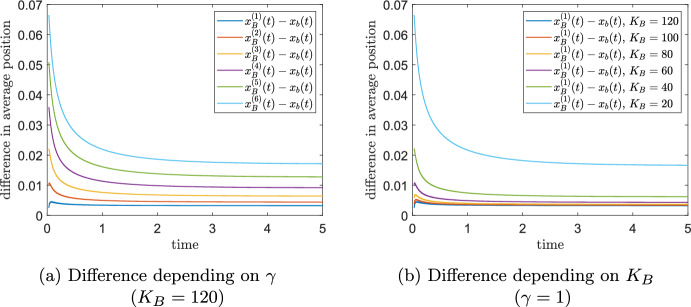
Time-dependent differences of average positions. **a** Differences between average positions $x_B^{(\gamma )}(t)$ of mgRDME, see (69), and average positions $x_b(t)$ of the ‘ground truth’ solution, see (62), for various $\gamma$ and fixed $K_B=120$. **b** Differences between average positions of the RDME (fixed $\gamma =1)$ and the ‘ground truth’ solution for various $K_A=K_B$. Rate constants given in (18) (Color figure online)

## Data Availability

In compliance with EPSRC’s open access initiative, the data in this paper is available from: https://doi.org/10.5281/zenodo.11072450.

## Appendix

### Time-Dependent Solution of the Brownian Dynamics Model

Let us consider that initially there are no molecules of *A* and *B* in the system, i.e.

47 $$\begin{aligned} a(x,0) \equiv b(x,0) \equiv 0. \end{aligned}$$

To solve the PDE (27) with initial condition (47) and boundary conditions (29), we apply the Fourier transform

$$\begin{aligned} \widehat{a}(\xi ,t) = \int _{-\infty }^{\infty } a(x,t) \, e^{-i \xi x} \, \text{ d } x. \end{aligned}$$

We get

$$\begin{aligned} \frac{\partial \widehat{a}}{\partial t} (\xi ,t) = - (D_A \, \xi ^2 + k_2) \, \widehat{a}(\xi ,t) + 2 k_1. \end{aligned}$$

Solving this ODE with the initial condition $\widehat{a}(\xi ,0)=0$, we obtain

48 $$\begin{aligned} \widehat{a}(\xi ,t) = \frac{2k_1}{D_A \, \xi ^2 +k_2} - \frac{2k_1 \, \exp \!\big [ - \left( D_A \, \xi ^2 +k_2\right) t \, \big ]}{D_A \, \xi ^2 +k_2}. \end{aligned}$$

Since the Fourier transform of the convolution is the product of Fourier transforms, we can calculate the Fourier inverse of (48) as follows

$$\begin{aligned} a(x,t) = \bar{a}(x) - \frac{\exp [-k_2 t]}{2 \sqrt{D_A t \pi }} \int _{-\infty }^{\infty } \bar{a}(y) \, \exp \!\left[ - \frac{(x-y)^2}{4 D_A t} \right] \, \text{ d } y, \end{aligned}$$

where $\bar{a}(x)$ is given by (33). In total we get

49 $$\begin{aligned} a(x,t) =&\frac{k_1}{\sqrt{D_A \, k_2}} \, \exp \!\left[ -\sqrt{\frac{k_2}{D_A}} \, |x|\right] \nonumber \\&- \frac{\exp [-k_2 t]}{2 \sqrt{D_A t \pi }} \int _{-\infty }^{\infty } \frac{k_1}{\sqrt{D_A \, k_2}} \, \exp \!\left[ -\sqrt{\frac{k_2}{D_A}} \, |y| \right] \, \exp \!\left[ - \frac{(x-y)^2}{4 D_A t} \right] \, \text{ d } y \end{aligned}$$

for the average number of *A*-molecules in the interval $[x,x+\text{ d }x]$ at time *t*.

### ’Ground Truth’ Solution: The Effect of the Boundary

Here, we derive the ‘ground truth’ solution of Sect. 2.3 for zero-flux boundary conditions. To do that, we will solve the ODEs (30–31) on the interval $[-L,L]$ with zero-flux boundary conditions

50 $$\begin{aligned} \frac{\text{ d } \bar{a}}{\text{ d } x} (-L) = \frac{\text{ d } \bar{a}}{\text{ d } x} (L) = \frac{\text{ d } \bar{b}}{\text{ d } x} (-L) = \frac{\text{ d } \bar{b}}{\text{ d } x} (L) = 0. \end{aligned}$$

Then the solution to the steady-state Eq. (30) is

$$\begin{aligned} \bar{a}(x) = \frac{k_1}{\sqrt{D_A \, k_2}} \, \exp \!\left[ -\sqrt{\frac{k_2}{D_A}} \, |x| \right] \frac{ 1 + \exp \!\left[ 2 \sqrt{\frac{k_2}{D_A}} \, \Big (|x|-L\Big ) \right] }{1-\exp [-2 L \sqrt{k_2/D_A}]}, \end{aligned}$$

which for $L\rightarrow \infty$ agrees with (33), independently of the parameter values.

### Second-Order Moments for Dimerization

Means and variances of the numbers of molecules of *A* and *B* for the dimerization system are plotted in Fig. 9, sampled from long-term simulations of the (standard) compartment-based model and of the particle-based Brownian dynamics model, choosing boxes of size $h=1/15$. We observe a good agreement of the two model types.

### Dynamics Prior to Steady State

To study the dynamics prior to steady state we consider the average positions of the particles in dependence on time. These investigations are done for the first-order morphogen gradient system introduced in Sect. 2.

#### Average Positions for ‘Ground Truth’ Solution

We define the moments

51 $$\begin{aligned} \langle 1 \rangle _a(t) := \int _0^\infty a(x,t) \, \text{ d }x \,, \qquad \qquad&\langle x \rangle _a(t) := \int _0^\infty x \, a(x,t) \, \text{ d }x \,, \end{aligned}$$

52 $$\begin{aligned} \langle 1 \rangle _b(t) := \int _0^\infty b(x,t) \, \text{ d }x, \qquad \qquad&\langle x \rangle _b(t) := \int _0^\infty x \, b(x,t) \, \text{ d }x \,. \end{aligned}$$

Multiplying Eqs. (27–28) by 1 and *x*, respectively, and integrating over $x \in [0,\infty )$, we obtain the ODEs

53 $$\begin{aligned} \frac{\text{ d } \langle 1 \rangle _a}{\text{ d } t}= &  - \, k_2 \, \langle 1 \rangle _a \, + \, k_1 , \end{aligned}$$

54 $$\begin{aligned} \frac{\text{ d } \langle x \rangle _a}{\text{ d } t}= &  D_A \, a(0,t) \, - \, k_2 \, \langle x \rangle _a , \end{aligned}$$

55 $$\begin{aligned} \frac{\text{ d } \langle 1 \rangle _b}{\text{ d } t}= &  k_2 \, \langle 1 \rangle _a \, - \, k_3 \, \langle 1 \rangle _b , \end{aligned}$$

56 $$\begin{aligned} \frac{\text{ d } \langle x \rangle _b}{\text{ d } t}= &  D_B \, b(0,t) \, + \, k_2 \, \langle x \rangle _a \, - \, k_3 \, \langle x \rangle _b . \end{aligned}$$

Substituting $x=0$ into Eq. (49), we have

57 $$\begin{aligned} a(0,t)= &  \frac{k_1}{\sqrt{D_A \, k_2}} \, - \, \frac{\exp [-k_2 \, t]}{\sqrt{D_A t \pi }} \int _{0}^{\infty } \frac{k_1}{\sqrt{D_A \, k_2}} \, \exp \!\left[ - \sqrt{\frac{k_2}{D_A}} \, y - \frac{y^2}{4 \, D_A \, t} \right] \, \text{ d } y \nonumber \\= &  \frac{k_1}{\sqrt{D_A \, k_2}} \, \text{ erf } \! \left[ \sqrt{k_2 \, t} \right] . \end{aligned}$$

Substituting (57) into Eq. (54), we get

$$\begin{aligned} \frac{\text{ d } \langle x \rangle _a}{\text{ d } t} = \frac{k_1 \, \sqrt{D_A}}{\sqrt{k_2}} \, \text{ erf } \! \left[ \sqrt{k_2 \, t} \right] \, - \, k_2 \, \langle x \rangle _a . \end{aligned}$$

Using the initial condition $\langle x \rangle _a(0)=0$, we obtain

58 $$\begin{aligned} \langle x \rangle _a(t)= &  \exp [-k_2 \, t] \int _0^t \exp \big [k_2 \, \tau \big ] \, \frac{k_1 \, \sqrt{D_A}}{\sqrt{k_2}} \, \text{ erf } \! \left[ \sqrt{k_2 \, \tau } \right] \, \text{ d } \tau \nonumber \\= &  \frac{k_1 \, \sqrt{D_A}}{k_2 \, \sqrt{k_2}} \, \text{ erf } \! \left[ \sqrt{k_2 \, t} \right] - \exp [-k_2 \, t] \, \frac{2 \, k_1 \, \sqrt{D_A \, t}}{k_2 \sqrt{\pi }}. \end{aligned}$$

Using the initial condition $\langle 1 \rangle _a(0)=0$ in Eq. (53), we obtain

59 $$\begin{aligned} \langle 1 \rangle _a(t) = \frac{k_1}{k_2} \, \big (1 - \exp [-k_2 t]\big ) \end{aligned}$$

for the average total number of *A*-particles at time *t*. Consequently, the average position $x_a(t)$ of *A*-particles as a function of time $t>0$ is

60 $$\begin{aligned} x_a(t) := \frac{\langle x \rangle _a(t)}{\langle 1 \rangle _a(t)} = \frac{ \sqrt{D_A \, \pi } \, \text{ erf } \! \left[ \sqrt{k_2 \, t} \right] - \exp [-k_2 \, t] \, 2 \, \sqrt{D_A k_2 \, t}}{\big (1 - \exp [-k_2 t]\big ) \, \sqrt{k_2 \, \pi }}. \end{aligned}$$

We note that this ratio is independent of our choice of the rate constant $k_1$.

Substituting (59) into Eq. (55), we get

$$\begin{aligned} \frac{\text{ d } \langle 1 \rangle _b}{\text{ d } t} = k_1 \, \big (1 - \exp [-k_2 t]\big ) \, - \, k_3 \, \langle 1 \rangle _b . \end{aligned}$$

Using the initial condition $\langle 1 \rangle _b(0)=0$, we obtain

61 $$\begin{aligned} \langle 1 \rangle _b(t)= &  \exp [-k_3 \, t] \int _0^t \exp \big [k_3 \, \tau \big ] \, k_1 \, \big (1 - \exp [-k_2 \tau ]\big ) \, \text{ d } \tau \nonumber \\= &  \frac{k_1}{k_3} \, + \, \frac{k_1 \, k_2 \, \exp [-k_3 \, t]}{(k_3-k_2) k_3} \, + \, \frac{k_1 \, \exp [-k_2 \, t]}{k_2-k_3} \, \end{aligned}$$

for the average total number of *B*-particles at time *t*. Substituting (58) into Eq. (56), we get

$$\begin{aligned} \frac{\text{ d } \langle x \rangle _b}{\text{ d } t} = D_B \, b(0,t) \, + \, \frac{k_1 \, \sqrt{D_A}}{\sqrt{k_2}} \, \text{ erf } \! \left[ \sqrt{k_2 t} \right] \,-\, \exp [-k_2 \, t] \, \frac{2 \, k_1 \, \sqrt{D_A \, t}}{\sqrt{\pi }} \, - \, k_3 \, \langle x \rangle _b . \end{aligned}$$

The average position $x_b(t)$ of *B*-particles at time *t* is given by

62 $$\begin{aligned} x_b(t)= \frac{\langle x \rangle _b(t)}{ \langle 1 \rangle _b(t)}. \end{aligned}$$

Fig. 10 shows the average total number of particles and their average positions depending on time for the ‘ground truth’ model.

#### Time-Dependent Solution for the Compartment-Based Model

In analogy to the densities *a*(*x*, *t*) and *b*(*x*, *t*) defined for the particle-based model, let $a_i(t)$ and $b_i(t)$ denote the average number of particles of species *A* and *B* in box *i* at time *t*, respectively. We set ${\textbf{a}}(t)=(a_i(t))_{i=1,...,K_A}$ and ${\textbf{b}}(t)=(b_i(t))_{i=1,...,K_B}$ and have

63 $$\begin{aligned} \frac{\text{ d }{\textbf{a}}}{\text{ d }t}&= \left( \frac{D_A}{h_A^2}S_A - k_2 I_A\right) {\textbf{a}} + k_1{\textbf{e}}_1, \end{aligned}$$

64 $$\begin{aligned} \frac{\text{ d }{\textbf{b}} }{\text{ d }t}&= \left( \frac{D_B}{h_B^2}S_B - k_3 I_B\right) {\textbf{b}} + \frac{k_2}{\gamma }M{\textbf{a}}, \end{aligned}$$

for $I_A, S_A\in {\mathbb R}^{K_A \times K_A}$, $I_B,S_B\in {\mathbb R}^{K_B \times K_B}$, ${\textbf{e}}_1 \in {\mathbb R}^{K_A}$ and $M\in \mathbb {R}^{K_B \times K_A}$ defined in Sect. 2.2. We note that the standard RDME is included by setting $K_A=K_B$. The ODEs (63)-(64) are the analogue to Eqs. (27–28) of the ‘ground truth’ dynamics. Together with ${\textbf{a}}(0)=0$ we obtain

65 $$\begin{aligned} {\textbf{a}}(t) = W_A^{-1} \left[ \exp \left( W_A t \right) - I_A \right] k_1{\textbf{e}}_1 \end{aligned}$$

for

$$\begin{aligned} W_A:=\frac{D_A}{h_A^2}S_A - k_2 I_A\in \mathbb {R}^{K_A\times K_A}. \end{aligned}$$

Moreover, using ${\textbf{b}}(0)=0$ and defining $W_B:=(D_B/h_B^2)S_B - k_3 I_B\in \mathbb {R}^{K_B\times K_B}$, we obtain (Perko 2013)

66 $$\begin{aligned} {\textbf{b}}(t)&= \frac{k_2}{\gamma } \int _0^t\exp (W_B (t-s)) M {\textbf{a}}(s) \, \text{ d }s\nonumber \\&{\mathop {=}\limits ^{\text {(A.19)}}}\frac{k_2}{\gamma } \int _0^t\exp (W_B (t-s)) M W_A^{-1} \left[ \exp \left( W_A s \right) - I_A \right] k_1{\textbf{e}}_1 \, \text{ d }s \end{aligned}$$

for the vector of time-dependent average numbers of *B*-particles in the boxes at time *t*. We have $\lim _{t\rightarrow \infty }{\textbf{a}}(t)=\overline{\textbf{A}}$ and $\lim _{t\rightarrow \infty }{\textbf{b}}(t)=\overline{\textbf{B}}$ for the steady-state solutions $\overline{\textbf{A}}$, $\overline{\textbf{B}}$ given in (16) and (24).

#### Average Positions for the Compartment-Based Model

In analogy to the moments defined in (51)-(52), we set

67 $$\begin{aligned}&\langle 1 \rangle _A(t) := \sum _{i=1}^{K_A}a_i(t) \,, \qquad \qquad \langle x \rangle _A(t) :=\sum _{i=1}^{K_A} (i-0.5)\,h_A \, a_i(t) \,, \end{aligned}$$

68 $$\begin{aligned}&\langle 1 \rangle _B(t) := \sum _{i=1}^{K_B} b_i(t), \qquad \qquad \langle x \rangle _B(t) := \sum _{i=1}^{K_B} (i-0.5)\,h_B\, b_i(t) \,. \end{aligned}$$

In the definitions of $\langle x \rangle _A(t)$ and $\langle x \rangle _B(t)$, we choose the centers $(i-0.5)h_A$ and $(i-0.5)h_B$ of the boxes for the spatial value, which is justified by the assumption that the particles in each box are spatially well mixed so that their positions follow a uniform distribution.

By summing Eqs. (63–64) over the discrete index *i* we obtain the same ODEs for the moments $\langle 1 \rangle _A(t)$ and $\langle 1 \rangle _B(t)$ as in the ‘ground truth’ model; see Eqs. (53) and (55). This means that the average total number of particles $\langle 1 \rangle _A(t)$ and $\langle 1 \rangle _B(t)$ is again given by Eqs. (59) and (61). We calculate the moments $\langle x \rangle _A(t)$ and $\langle x \rangle _B(t)$ according to their definition in (67)-(68), using the solutions ${\textbf{a}}(t)$ and ${\textbf{b}}(t)$ given in (65) and (66). Given $\gamma =K_B/K_A$, the mean positions $x_A^{(\gamma )}(t)$ and $x_B^{(\gamma )}(t)$ of *A* and *B* molecules at time *t* are

69 $$\begin{aligned} x_A^{(\gamma )}(t)= \frac{\langle x \rangle _A(t)}{\langle 1 \rangle _A(t)}, \quad \quad x_B^{(\gamma )}(t)= \frac{\langle x \rangle _B(t)}{\langle 1 \rangle _B(t)}, \end{aligned}$$

in analogy to (60) and (62), respectively.

Figure 11 shows the time-dependent differences between the average positions $x_B^{(\gamma )}(t)$ of the mgRDME and the average positions $x_b(t)$ of the ‘ground truth’ solution for various $\gamma$ and $K_B$. We observe that for every time $t>0$, the error increases with the ratio $\gamma =K_B/K_A$ for fixed $K_B=120$, see Fig. 11a. Similarly, the error increases with decreasing values of $K_B$, see Fig. 11b. In contrast to the results from Sect. 2.4, the approximation quality is here more robust against changes in $K_B$; this can be explained by the fact that the reference value is given by the average positions and not by the spatial distribution of molecules of *B*.

The mean position $x_B^{(\gamma )}(t)$ of the compartment-based model always exceeds the mean position $x_b(t)$ of the ‘ground truth’ solution. Especially for the initial time phase (small *t*), the difference is large. This is due to the fact that the production of molecules of *A* is located at position $x=0$. In the mgRDME, the newly produced particles immediately get well mixed in the first box $[0,h_A]$, while in the ‘ground truth’ model it takes some time for the particles to spread out in space. The mixing in the mgRDME implies a shift of the average to the right, which has a larger effect especially in the beginning, where the population is small and mostly located near the origin. This effect, which also propagates to *B* via conversion, can be interpreted as an artificial dispersion induced by the mgRDME.

## Footnotes

1 The values result from setting the related reaction rate equations to zero, i.e. from solving $k_1-k_2\bar{A}_{\text {total}}=0$ and $k_2\bar{A}_{\text {total}}-k_3\bar{B}_{\text {total}}=0$. We note that this approach is only applicable to first-order reaction systems.
